# Oral health and use of dental services in different stages of adulthood in Norway: a cross sectional study

**DOI:** 10.1186/s12903-021-01626-9

**Published:** 2021-05-13

**Authors:** Elin Hadler-Olsen, Birgitta Jönsson

**Affiliations:** 1 The Public Dental Health Service Competence Center of Northern Norway, Tromsö, Norway; 2 Department of Medical Biology, Faculty of Health Sciences, UiT the Arctic University of Norway, Tromsö, Norway; 3 Department of Periodontology, Institute of Odontology, The Sahlgrenska Academy, University of Gothenburg, Gothenburg, Sweden

**Keywords:** Oral health, Dental caries, Number of teeth, Age-stratified analyses, General health, Financial barrier

## Abstract

**Background:**

Socioeconomic status and oral health care habits may change throughout adult life. This calls for age-stratified analyses of oral health in the adult population to uncover differences that could be of importance for organizing adequate oral health care services. The aim of the present study was to describe self-reported oral health in different age groups in a general adult population in Norway, and to explore associations between self-reported oral health and age groups, sociodemographic factors, use of dental services, number of teeth and dental caries.

**Methods:**

We used data from a cross-sectional study of almost 2000 Norwegian adults, 20–79 years old. The study included both a structured questionnaire and a clinical examination to assess sociodemographic variables, use of dental services, self-reported oral and general health as well as dental caries and number of teeth. For analysis, the participants were divided into three age groups: young adults (20–29 years), middle-aged adults (30–59 years), and senior adults (60 years and older). Differences among groups were analyzed by cross-tabulation, and logistic regression analyses were used to assess associations between variables.

**Results:**

Forty-eight percent of the participants rated their oral health as good. Almost half of the participants had at least one carious tooth, with the highest caries prevalence among the young adults. To be caries free was strongly associated with reporting good oral health among the young and middle-aged adults. One third of the senior adults had fewer than 20 teeth, which was associated with reporting moderate or poor oral health. Less than half of the young adults reported regular use of dental services, and 40% of them had postponed dental visits for financial reasons during the past 2 years. Regardless of age group, having to postpone dental visits for financial reasons or having poor-to-moderate general health were associated with high odds for reporting moderate or poor oral health.

**Conclusions:**

That there were important age-group differences in self-reported and clinical measures of oral health and in the use of dental health services demonstrates the importance of age-stratified analyses in oral health research. Many adults, especially among the young, faced financial barriers for receiving dental health services, which was associated with poorer self-reported oral health. This argues for a need to revisit the financing of oral health care for adults in Norway.

## Background

Over the past 50 years, there has been a marked improvement in clinical measures of oral health [1, 2], partly due to the wide use of fluoridated toothpaste and a stronger focus on disease prevention in the dental health services of most Western countries. However, many studies have found rather weak associations between clinical and self-reported measures of oral health [3–5]. Thus, when evaluating the oral health of a population, it is important to use both clinical and self-perceived measures.

The regular use of dental health services is associated with good oral health [6–9], suggesting that it is essential to ensure that dental health services are available to the population. Oral health and the use of oral health services may vary throughout life as attitudes, financial means and general health change. In Norway, oral health services for children and adolescents are almost completely covered by the public, whereas from the age of 21, they are paid out-of-pocket, with few exceptions. This coincides with a period in life when most young adults in Norway move out of their childhood home, are studying or about to establish their professional careers, and have a low or irregular income. Between 30 and 60 years of age, life is often more stable, and most people are working and have a regular income. People older than 60 years did not have the benefit of fluoridated toothpastes until they reached adult age, and they may have had less regular dental checkups during their childhood and adolescent years because the public dental health services at that time were less developed. Furthermore, with increasing age, general health usually deteriorates, and this may subsequently affect the oral health and the ability to use oral health services. The change in oral health over the past decades, along with differences in socioeconomic status, and general health in different phases of life, calls for a need to study oral health and use of oral health services in different age groups of the adult population. Age-stratified analyses can uncover nuances that are of importance for organizing oral health services that best meet people’s needs throughout their life course. Thus, the aim of the present study was to describe self-reported oral health and its associations in different age groups of adults in a general population in Norway.

## Methods

### Study design and participants

We used data from the Oral Health in Northern Norway (TOHNN) study, a cross-sectional study of an adult population in Troms County in Northern Norway. The study protocol including power calculations, selection and invitation procedures and calibration of examiners, has been described in detail previously [10]. In brief, we invited a simple random sample of 2901 persons (2.6% of the residents) between 20 and 79 years in Troms County to participate in the study, of whom 1986 (68.5%) both answered a questionnaire and completed a dental examination. The dental examinations were performed by eleven calibrated examiner teams (dentist with assistant nurse) in five different dental clinics between October 2013 and November 2014. In the present study, edentulous participants were excluded (n = 50). The study was approved by The Regional Committee for Medical and Health Research Ethics of Northern Norway (2013/348/REK Nord).

### Variables

*Outcome variable* Self-reported oral health was assessed by one question with five response options from I (very poor) to V (very good). For cross-tabulation analyses, we trichotomized the responses as (1) poor (option I and II); (2) moderate (option III); and (3) good (option IV and V). When used as a dependent variable in regression analyses, we dichotomized self-reported oral health into (1) good (options IV and V) versus moderate or poor (options I, II and III).

*Exposure variable* Age was categorized into three groups: (1) young adults (20–29 years), (2) middle-aged adults (30–59 years), and (3) senior adults (60 years and older).

*Covariates/confounders* Gender (male; female), municipality size (large city (> 50,000); small town (10,000–50,000); village (< 10,000)) and highest completed education (secondary school; high school; college/university) were assessed with one question each and used without reclassification in the analyses. Tooth brushing frequency, dental attendance, reason for not having regular dental check-ups, and if they had postponed dental visits due to economy during the past 2 years were assessed with one question each. For analyses, we dichotomized tooth-brushing frequency into (1) less than twice a day; and (2) tooth brushing twice a day or more frequent. Similarly, dental attendance was dichotomized into (1) regular visits at least every second year; and (2) longer intervals. The options for not having regular dental check-ups were divided into four categories: (1) no need or wish to see a dentist/dental hygienist; (2) availability, which included distance to clinic, long waiting time for an appointment, no designated dentist/dental hygienist and not been summoned; (3) economy; and (4) anxiety or discomfort. Respondents with unspecified reason (n = 53) were excluded. The question about “Postponed dental visits for financial reasons during the past 2 years” had two response options: (1) yes, and (2) no. Self-reported general health was assessed by one question with five response options from I (very poor) to V (very good). For cross-tabulation analyses we trichotomized the responses as: (1) poor (option I and II); (2) moderate (option III); and (3) good (option IV and V). Due to few respondents reporting poor general health, the variable was dichotomized for regression analyses: (1) poor or moderate (options I, II and III) versus good (options IV and V).

The number of teeth (wisdom teeth excluded) was assessed by clinical and radiographic examination (panoramic x-rays) and were divided into two categories for analyses: (1) 1–19 teeth, and (2) ≥ 20 teeth. The method for assessing dental caries in the TOHNN-study has been described previously [11]. In brief, all teeth except for the third molars were examined clinically and radiographically on bite-wing radiographs, by 11 dental teams. A five-grade diagnostic scale was used to register severity of the caries, where grades 3–5 denoted caries involving the dentine.

Prior to the study, all examiners were trained and calibrated towards a gold standard regarding the diagnostic criteria and examination procedures. Two calibration tests were conducted during the study period, giving an acceptable agreement (Cohen’s kappa (j) median value 0.73 and 0.77). We divided the number of carious teeth (grade 3–5 involving dentine) into the three categories of 0 carious teeth, 1–3 carious teeth and ≥ 4 carious teeth.

### Statistical analyses

We used the Statistical Package for Social Sciences (SPSS) for Windows version 26 (IBM corporation, Armonk, NY, USA) for statistical analyses. Means are presented ± the standard deviation. Differences among groups were assessed by cross-tabulation, and Pearson’s Chi-square test was used to test the statistical significance of the observed differences. Univariate and multivariate logistic regression analyses with forced entry used with self-assessed oral health as the dependent variable, age group as the exposure variable, and the other variables listed in Table 1 as covariates. The reason for not attending regular dental visits was excluded from multivariate analyses because it was strongly correlated with two of the other variables (regular dental visits and postponed dental visits for financial reasons in the past 2 years). The number of teeth was not included in regression analyses for young and middle-aged adults due to few respondents having fewer than 20 teeth in these age groups. The results are presented as odds ratios (OR) with 95% confidence intervals (95% CI).

**Table 1:** Sociodemographic characteristics, use of oral health services and oral health measures in the different age groups

	All n (%)	20–29 years n (%)	30–59 years n (%)	≥ 60 years n (%)	Chi-square *P*
Gender					
Male	945 (48.9)	132 (41.9)	545 (48.8)	268 (53.5)	0.006
Female	987 (51.1)	183 (58.1)	571 (51.2)	233 (46.5)	
Municipality size					
> 50,000	867 (44.9)	158 (50.2)	513 (46.0)	196 (39.1)	0.002
10,000–50 000	598 (31.0)	82 (26.0)	359 (32.2)	157 (31.3)	
< 10,000	467 (24.2)	75 (23.8)	244 (21.9)	148 (29.5)	
Education					
Secondary school	281 (14.5)	19 (6.0)	103 (9.2)	159 (31.7)	< 0.001
High school	853 (44.2)	188 (59.7)	458 (41.0)	207 (41.3)	
University/college	798 (41.3)	108 (34.3)	555 (49.7)	135 (26.9)	
Self-reported oral health					
Good	1414 (73.3)	260 (82.5)	846 (76.1)	308 (61.5)	< 0.001
Moderate	443 (23.0)	48 (15.2)	227 (20.4)	168 (33.5)	
Poor	71 (3.7)	7 (2.2)	39 (3.5)	25 (5.0)	
Tooth brushing frequency					
< 2/day	537 (27.8)	100 (31.7)	275 (24.6)	162 (32.3)	0.001
≥ 2/day	1395 (72.2)	215 (68.3)	841 (75.4)	339 (67.7)	
Dental visits					
≥ every 2nd year	1270 (66.4)	150 (47.8)	754 (68.1)	366 (74.2)	< 0.001
< every 2nd year	644 (33.6)	164 (52.2)	353 (31.9)	127 (25.8)	
Postponed dental visits for financial reasons past 2 years					
No	1549 (80.4)	189 (60.0)	897 (80.6)	463 (92.8)	< 0.001
Yes	378 (19.6)	126 (40.0)	216 (19.4)	36 (7.2)	
Why irregular dental visits					
No need/wish	208 (28.3)	48 (26.2)	100 (24.8)	60 (40.3)	< 0.001
Availability	149 (20.2)	34 (18.6)	83 (20.5)	32 (21.5)	
Economy	226 (30.7)	73 (39.9)	117 (29.0)	36 (24.2)	
Anxiety	153 (20.8)	28 (15.3)	104 (25.7)	21 (14.1)	
Self-reported oral health					
Good	933 (48.4)	139 (44.1)	590 (53.0)	204 (40.9)	< 0.001
Moderate	742 (38.5)	141 (44.8)	386 (34.7)	215 (43.1)	
Poor	252 (13.1)	35 (11.1)	137 (12.3)	80 (16.0)	
Carious teeth					
0	1016 (52.7)	135 (42.9)	570 (51.2)	311 (62.3)	< 0.001
1–3	741 (38.5)	128 (40.6)	456 (41.0)	157 (31.5)	
> 3	170 (8.8)	52 (16.5)	87 (7.8)	31 (6.2)	
Number of teeth					
≥ 20	1742 (90.5)	312 (99.4)	1097 (98.7)	333 (66.9)	< 0.001
< 20	182 (9.5)	2 (0.6)	15 (1.3)	165 (33.1)	

n = number in the group

Chi-square *P* = two-sided significance of difference between age groups assessed by the Pearson Chi-square test

## Results

This study included 1927 participants (51% women) from 20 to 79 years of age, (mean 47.5 ± 0.3), of whom 16% were 20–29-year-olds, 58% were 30–59-year-olds and 26% were 60 years or older.

### Sociodemographic characteristics, oral health behavior and oral health in different age groups

Table 1 summarizes sociodemographic characteristics, use of oral health services and oral health measures in the different age groups. Sociodemographic characteristics differed significantly by age groups, whereby young adults had the highest proportion living in a large city, but middle-aged had the highest proportion with a university degree. Three-quarters of participants rated their general health as good, with this ranging from 83% of the young adults to 62% of the seniors.

The percentage of respondents who were seeing a dentist or dental hygienist regularly at least every second year ranged from just under half of the young adults to three-quarters of the seniors. Respondents who did not have regular dental check-ups at least every second year were asked for the most important reason for irregular dental visits. For the young and middle-aged adults, financial reasons were most common, whereas no need or wish to see a dentist was the most common reason for senior adults. The proportion that had postponed dental visits for financial reasons during the past 2 years differed markedly with age, ranging from 40% of the young adults to 7% of the seniors. Young and senior adults reported less frequent tooth brushing than did the middle-aged.

Self-assessed oral health was rated as good among 48% of the respondents, whereas 39% and 13% reported moderate or poor oral health, respectively. The proportion of respondents reporting poor oral health was slightly higher in the older age groups than in the young adults. However, there was a lower proportion among young adults reporting good oral health than among the middle-aged adults. Fifty-three percent of the participants had no dental caries. The young adults had by far the highest caries prevalence, with 43% having no caries and 16% having more than three carious teeth. The mean number of teeth for the participants was 25.1 ± 0.1 and the median was 27. Ten percent of the respondents had fewer than 20 teeth, ranging from 1% in young and middle-aged adults to 33% of senior adults.

### Regression analyses

Regression analyses were performed with self-reported oral health dichotomized as good versus moderate or poor oral health as dependent variable (Table 2).

**Table 2:** Logistic regression analyses for good versus moderate or poor self-reported oral health

	Good self-reported oral health versus moderate/poor			
	All participants	20–29 years	30–59 years	≥ 60 years
	Adjusted OR (95% CI)	Adjusted OR (95% CI)	Adjusted OR (95% CI)	Adjusted OR (95% CI)
Gender				
Male	Ref	Ref	Ref	Ref
Female	**1.39 (1.12–1.73)**	**1.95 (1.12–3.37)**	1.33 (0.99–1.76)	1.26 (0.81–1.96)
Municipality size				
> 50,000	**1.46 (1.12–1.91)**	0.84 (0.43–1.63)	**1.53 (1.07–2.20)**	**1.87 (1.12–3.13)**
10,000–50,000	1.07 (0.81–1.42)	0.96 (0.45–2.04)	1.33 (0.91–1.95)	0.75 (0.44–1.28)
< 10,000	Ref	Ref	Ref	Ref
Education				
Secondary school	Ref	Ref	Ref	Ref
High school	0.85 (0.61–1.19)	1.03 (0.32–3.37)	0.82 (0.49–1.37)	0.76 (0.45–1.28)
University/college	1.36 (0.97–1.93)	1.53 (0.45–5.21)	1.34 (0.81–2.23)	1.17 (0.65–2.10)
General health				
Good	**4.01 (3.11–5.17)**	**2.95 (1.38–6.50)**	**4.38 (3.11–6.17)**	**3.69 (2.33–5.85)**
Moderate/poor	Ref	Ref	Ref	Ref
Tooth brushing				
< 2/day	Ref	Ref	Ref	Ref
≥ 2/day	1.28 (0.99–1.64)	1.68 (0.92–3.08)	**1.50 (1.07–2.09)**	0.83 (0.51–1.35)
Dental visits				
≥ every 2nd year	**2.35 (1.86–2.97)**	**1.88 (1.11–3.21)**	**2.35 (1.73–3.19)**	**3.43 (1.92–6.12)**
< every 2nd year	Ref	Ref	Ref	Ref
Postponed dental visits for financial reasons				
No	**2.81 (2.10–3.77)**	**2.89 (1.62–5.14)**	**3.07 (2.09–4.51)**	**4.69 (1.43–15.33)**
Yes	Ref	Ref	Ref	Ref
Carious teeth				
0	**3.20 (2.07–4.94)**	**4.41 (1.84–10.53)**	**3.57 (1.93–6.59)**	2.31 (0.89–5.96)
1–3	**2.11 (1.35–3.26)**	2.25 (0.95–5.33)	**2.39 (1.29–4.43)**	1.57 (0.59–4.17)
> 3	Ref	Ref	Ref	Ref
Number of teeth				
≥ 20	**2.58 (1.70–3.91)**	^a^	^a^	**2.20 (1.34–3.62)**
< 20	Ref			Ref
Model summary				
Nagelkerke R^2^	R^2^ = 0.312	R^2^ = 0.308	R^2^ = 0.321	R^2^ = 0.307
Omnibus test coefficient	*P* < 0.001	*P* < 0.001	*P* < 0.001	*P* < 0.001
Hosmer and Lemeshow	*P* = 0.266	*P* = 0.349	*P* = 0.141	*P* = 0.568

*OR* odds ratio, *95% CI* 95% confidence interval. Numbers in bold indicates two-sided *P* value < 0.05

^a^Number of teeth excluded in regression analyses due to few respondents with < 20 teeth

Across age groups, reporting good general health and good oral health was strongly associated. Among young adults, women had two times higher odds than men of reporting good oral health, otherwise there were no significant gender differences. Living in a large city was associated with reporting good oral health among the middle-aged and seniors. Education was not significantly associated with self-reported oral health in any age group.

#### Oral health behavior: dental visits and tooth brushing

Tooth brushing at least twice a day was associated with reporting good oral health among middle-aged, but not among young- or senior adults. Regardless of age groups, regular dental visits and postponing dental visits due to financial reasons in the past 2 years were consistently associated with self-assessed oral health, with stronger associations for the latter. In univariate analyses those who reported no need or wish or availability as the most important reasons for irregular dental visits had much higher odds of reporting good oral health than had those reporting financial means or anxiety as the most important reason (OR = 1.00 and OR = 0.61for no need or wish and availability, respectively, versus OR = 0.18 and OR = 0.23 for financial reasons and anxiety, respectively).

#### Dental caries and number of teeth

Among young and middle-aged adults, good self-reported oral health was strongly associated with being caries-free, whereas having 20 teeth or more was associated with reporting good oral health among seniors.

## Discussion

The present study describes self-assessed and clinical measures of oral health in a large, age-stratified adult population from Troms County in Norway, and their associations with each other as well as to use of dental services and sociodemographic variables. As the study is cross-sectional, cause and effect relationships cannot be determined. Despite the marked improvement in clinical oral health over the past 50 years, fewer than 50% of the respondents in our study rated their oral health as good. Young adults had a lower proportion reporting good oral health than middle-aged, whereas senior adults had the highest proportion reporting poor oral health. Self-reported general health was over-all better than self-reported oral health, but the two measures were associated in all age groups. Young adults had the highest prevalence of dental caries, and seniors had by far the most tooth loss, with one third having fewer than 20 teeth. Self-reported oral health was strongly associated with dental caries among young and middle-aged adults and with tooth loss among seniors. Fewer than 50% of the young adults reported regular dental visits, with lack of money as the most important reason for irregular attendance. To have irregular dental visits and postpone dental visits due to financial reasons was associated with moderate or poor oral health regardless of age groups.

More participants in the present study than in two previous studies of adults in Norway [12, 13] reported poor oral health. In a representative national cohort of Norwegian adults, 71% rated their oral health as good, and 7% reported poor oral health [12]. Another study assessed self-reported oral health in three different counties in Norway, including Troms County [13], and found better self-reported oral health over-all than in the present study. These previous studies did not report age-stratified data and did not include a clinical examination, only a questionnaire sent by mail. The present study included a free dental examination and may thereby have encouraged invited persons with limited financial means and irregular dental visiting habits to participate, thereby contributing to the high proportion of participants reporting poor oral health.

Few studies have examined age-differences in self-reported oral health in adults. One previous study assessed self-reported oral health in different adult age groups in Australia and America, but with a different scale, making a direct comparison of the findings difficult [14]. This study found that the proportion of respondents reporting excellent or very good oral health was lower in older age groups in both populations, whereas proportion of respondents reporting poor or very poor oral health was higher in the 18–34-year age group than in the 35–44-year age group in the American population. In the present study, self-reported oral health corresponded fairly well with clinical oral health measures—dental caries in young and middle-aged adults and tooth loss in senior adults. However, many factors can affect how different age groups rate their health. Several studies have found that satisfaction with life in general is stable or even increases with age, although many objective measures related to health and quality of life are deteriorating. This is sometimes labeled the subjective well-being or disability paradox, and has been explained by factors such as improved strategies to cope with stress, improved ability to regulate emotions, and reduced aspirations and comparison standards in elderly, causing a smaller discrepancy between aspirations and achievements with increasing age [15–18]. These psychological factors may also affect the self-rating of oral health and contribute to the impression of a discordance between the severity of clinical oral health measures and self-reported oral health in different age groups.

Regular dental visiting has been shown to be associated with better clinical and subjective measures of oral health [6, 7, 11]; this is consistent with our findings. As previously reported, the participants who had regular dental check-ups had a lower prevalence of dental caries than those who were irregular dental visitors [7, 11]. There can be many reasons for irregular dental visiting, and they may differ among age groups as priorities, financial resources and life situation in general change. For senior adults, the most common reason for irregular dental visits was no wish or perceived need to see a dentist. Participants giving this reason had better self-reported oral health than had those giving other reasons for irregular visits. For young and middle-aged adults, financial reasons were the most common cause of irregular dental visiting, and almost 40% of the young adults and 20% of the middle-aged had postponed dental visits due to a lack of money during the past 2 years. In univariate analyses, refraining from regular dental visits due to financial reasons or anxiety was more strongly associated with poor-to-moderate self-reported oral health than having availability as the main obstacle. This suggests that being poor or anxious may be restrictions that are more difficult to over-come than availability if faced with a strong subjective need for help. To postpone dental visits due to financial reasons was independently associated with poorer self-assessed oral health, regardless of age. Our findings suggests that financial barriers for dental health care have a strong impact on self-reported oral health.

## Conclusion

The important age-group differences in self-reported and clinical oral health and in the use of the dental health services demonstrate the importance of age-stratified analyses in oral health research. Many adults, especially among the young, reported financial barriers to receiving oral health services. This argues for a need to revisit the out-of-pocket financing of oral health care for adults in Norway to make them available to everyone.

## Data Availability

The datasets generated and/or analyzed during the current study are not publicly available due EU GPDR regulation, we cannot make the data into so called “open access data”, but the data are available from the corresponding author on reasonable request.

## Abbreviations

DMFT: Decayed, missed, filled teeth index
TOHNN: The Oral Health in Northern Norway study
CI: Confidence interval
OR: Odds ratio
